# Demography of an ice-obligate mysticete in a region of rapid environmental change

**DOI:** 10.1098/rsos.220724

**Published:** 2022-11-02

**Authors:** L. Pallin, K. C. Bierlich, J. Durban, H. Fearnbach, O. Savenko, C. S. Baker, E. Bell, M. C. Double, W. de la Mare, J. Goldbogen, D. Johnston, N. Kellar, R. Nichols, D. Nowacek, A. J. Read, D. Steel, A. Friedlaender

**Affiliations:** ^1^ Department of Ecology and Evolutionary Biology, University of California Santa Cruz, Ocean Health Building, 115 McAllister Way, Santa Cruz, CA 95060, USA; ^2^ Institute for Marine Science, University of California Santa Cruz, Ocean Health Building, 115 McAllister Way, Santa Cruz, CA 95060, USA; ^3^ Department of Ocean Sciences, University of California Santa Cruz, Ocean Health Building, 115 McAllister Way, Santa Cruz, CA 95060, USA; ^4^ Division of Marine Science and Conservation, Nicholas School of the Environment, Duke University Marine Laboratory, 135 Duke Marine Lab Road, Beaufort, NC 28516, USA; ^5^ Marine Mammal Institute, Department of Fisheries, Wildlife, & Conservation Sciences, Oregon State University, Hatfield Marine Science Center, 2030 SE Marine Science Drive, Newport, OR, USA; ^6^ SeaLife Response, Rehabilitation, and Research, Des Moines, WA 98198, USA; ^7^ National Antarctic Scientific Center of Ukraine, 16 Taras Shevchenko Blvd, 01601, Kyiv, Ukraine; ^8^ Ukrainian Scientific Center of Ecology of the Sea, 89 Frantsuzsky Blvd, 65009, Odesa, Ukraine; ^9^ Australian Antarctic Division, 203 Channel Highway, Kingston, Tas 7050, Australia; ^10^ Hopkins Marine Station, Department of Biology, Stanford University, 120 Ocean View Blvd, Pacific Grove, CA 93950, USA; ^11^ Marine Mammal and Turtle Division, Southwest Fisheries Science Center, National Marine Fisheries Service, National Oceanic and Atmospheric Administration, 8901 La Jolla Shores Drive, La Jolla, CA 92037, USA

**Keywords:** antarctic minke whale, progesterone, demography, pregnancy, climate change, photogrammetry

## Abstract

Antarctic minke whales (*Balaenoptera bonaerensis*, AMW) are an abundant, ice-dependent species susceptible to rapid climatic changes occurring in parts of the Antarctic. Here, we used remote biopsy samples and estimates of length derived from unoccupied aircraft system (UAS) to characterize for the first time the sex ratio, maturity, and pregnancy rates of AMWs around the Western Antarctic Peninsula (WAP). DNA profiling of 82 biopsy samples (2013–2020) identified 29 individual males and 40 individual females. Blubber progesterone levels indicated 59% of all sampled females were pregnant, irrespective of maturity. When corrected for sexual maturity, the median pregnancy rate was 92.3%, indicating that most mature females become pregnant each year. We measured 68 individuals by UAS (mean = 8.04 m) and estimated that 66.5% of females were mature. This study provides the first data on the demography of AMWs along the WAP and represents the first use of non-lethal approaches to studying this species. Furthermore, these results provide baselines against which future changes in population status can be assessed in this rapidly changing marine ecosystem.

## 1. Introduction

The status of biological populations can be inferred by monitoring changes in parameters such as abundance, fecundity, mortality and age structure [1]. In the absence of direct estimates of abundance, demographic metrics may serve as indicators of population growth, viability and response to environmental changes [2–5]. In long-lived species, changes in demography are more likely to be detected over shorter timescales compared with changes in abundance, particularly for large populations such as baleen whales, where estimating abundance is difficult and often imprecise due to their marine distribution and cryptic behaviour.

Among long-lived large vertebrates, the effects of climate change have been well-studied in ungulates, primarily focused on how climate variability impacts births, survival and age structure [2,6,7]. For example, in 21 populations of woodland caribou, colder temperatures and increasing snowfall increase juvenile recruitment and population growth [8]. However, as climate anomalies (i.e. warmer temperatures, freeze–thaw events) become more common and larger in magnitude, an overall decrease in habitat availability and forage quality, an increase in adult energy expenditure, a decrease in pregnancy rates and an increase in predation risk have been observed [8–10]. Similar climate-driven shifts in demography and dynamics have been shown in elk [7], pronghorn antelope [11], moose, owls and wolves [12].

Understanding the impacts of climate-driven changes on polar species is particularly important given the rapid changes occurring at the poles in both marine and terrestrial ecosystems [13,14]. Antarctic minke whales (*Balaenoptera bonaerensis,* AMW) are an abundant ice-dependent species found year-round in the Antarctic [15,16]. They have a circumpolar distribution, probably breeding between 7° and 35° S [17]. AMWs have a strong affinity for ice-covered regions or sheltered bays, especially in areas with high densities of krill, their preferred prey [15,18–23]. AMWs are well-adapted to feed on krill under sea ice [24] and use sea-ice habitat to avoid predation by killer whales [25,26]. Due to the logistical challenges of studying pagophilic animals, particularly a cryptic marine species like AMWs, little is known about their life history or demography [27,28].

The extent of annual sea ice appears to be constant or expanding around most of the Antarctic, but the Western Antarctic Peninsula (WAP) is experiencing some of the most pronounced loss of sea ice in polar regions [29]. An estimated 1500 AMWs (95% CI: 1221–1953; [20]), inhabit the continental shelf waters around the WAP, which has experienced significant warming [30] and substantial reductions in the extent and duration of sea ice cover over the last 50 years [31]. These changes have resulted in a cascade of effects throughout the WAP ecosystem, and are probably impacting the demography, behaviour and ecology of AMWs [32]. Recent advances in both molecular ecology and unoccupied aircraft systems (UAS) technology allow us to study the demography of these whales using non-lethal techniques. In this study, we analysed skin and blubber biopsy samples and UAS-derived measurements of individual AMWs around the WAP to characterize, for the first time, their maturity, sex ratio and pregnancy rates. Our findings help fill key data gaps on the demographic structure and population trajectory of AMWs in this rapidly changing region.

## 2. Methods

### 2.1. Biopsy collection

**Figure 1.**
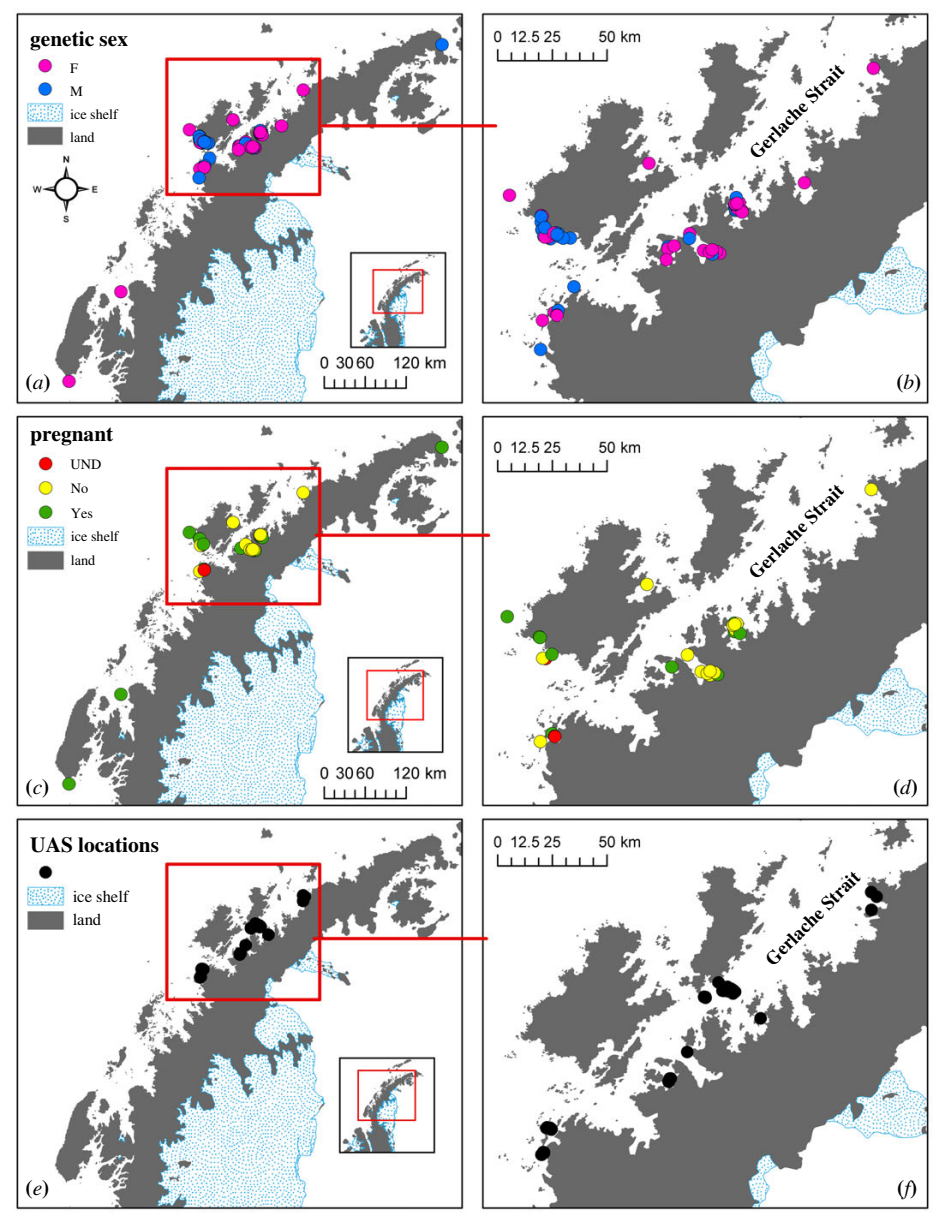
Genetic sex of Antarctic minke whales (AMWs) sampled along the Western Antarctic Peninsula (WAP) (*a*) and in the Gerlache Strait and adjacent bays (*b*), pregnancy status of female AMWs sampled along the WAP (*c*) and in the Gerlache Strait and adjacent bays (*d*), and location of AMWs imaged along the WAP (*e*) and in the Gerlache Strait and adjacent bays (*f*).

We collected skin and blubber biopsy samples from AMWs during the 2013–2020 austral summer and autumn (January–July) field seasons using standard techniques [33]. Samples were collected opportunistically during dedicated research cruises or from platforms of opportunity, including ecotour vessels, in the nearshore waters of the WAP (figure 1). We used a crossbow to project modified bolts and 40 mm stainless steel biopsy tips (CetaDart) to obtain samples from a distance of 10–30 m, targeting the area of the body below the dorsal fin. All age and sex classes of AMWs were sampled, except dependent calves. Samples were stored frozen whole at −20°C until used for analysis. Electronic supplementary material, data, (including location, group size) were recorded at every biopsy event.

### 2.2. DNA profiling

We used standard molecular methods to identify the sex of individuals from DNA extracted from biopsies [34,35]. We used a standard DNA profile, including sex-specific markers and microsatellite genotypes, to identify individual whales. Genomic DNA was extracted from the skin–blubber interface using a commercially available kit (Dneasy 96 Blood & Tissue Kit, Qiagen, Hilden, Germany). The sex of each sampled whale was determined by amplification of sex-specific markers following the protocols of Gilson *et al*. [36]. Results were compared with controls for a known male and female using gel electrophoresis. Sex ratios, depicted as the ratio of males to females (M : F), were calculated for the entire dataset and for specific sampling years.

Samples were genotyped using 10 previously published microsatellite loci to resolve the identity of each sampled whale and remove potential replicate samples (table 1) [37–40]. Alleles were sized and binned using the software program Genemapper v. 3.7 (Applied Biosystems). The total number of amplified loci for a given sample was considered as an added quality control threshold. Given the estimated probability of identity for these loci from previous studies [41], we considered samples matching at a minimum of seven loci to be recaptures of the same individual. Samples with fewer than seven microsatellite loci were repeated or excluded. The expected probability of identity (*P*_ID_; the probability that two individuals are drawn at random from a population will have the same genotype by chance) for each locus was calculated in GenAlEx v. 6.5 [42]. Cervus 3.0.7 [43] was used to compute the number of alleles (*K*), observed and expected heterozygosity (*H*_O_ and *H*_E_), and the probability of identity for all individual matches.

**Table 1.** Summary of microsatellite loci used for individual identification of Antarctic minke whales along the Western Antarctic Peninsula. The number of alleles, observed (*H*_O_), and expected (*H*_E_) heterozygosity was calculated using *cervus 3.0.1.* The expected probability of identity (*P*_ID_) of each locus was calculated with the program *GenAlEx v6.5*.

locus	source	label	MgCl_2_ (mM)	size range (bp)	no. of alleles	*H*_E_	*H*_O_	*P*_ID_
Ev1	Valsecchi & Amos [37]	NED	4	114–155	13	0.844	0.841	0.043
Ev37	Valsecchi & Amos [37]	NED	3.5	184–220	16	0.915	0.380	0.014
Ev104	Valsecchi & Amos [37]	FAM	2.5	126–160	16	0.894	0.918	0.021
GATA98	Palsbøll *et al.* [39]	NED	2.5	80–108	8	0.772	0.758	0.082
GT23	Berube *et al.* [38]	VIC	2.5	88–120	16	0.889	0.922	0.022
GT211	Palsbøll *et al.* [39]	FAM	2.5	80–114	14	0.887	0.921	0.023
GT509	Berube *et al.* [38]	HEX	2.5	179–217	31	0.952	0.967	0.004
GT575	Berube *et al.* [38]	FAM	1.5	129–161	15	0.906	0.872	0.016
rw4–10	Waldick *et al.* [40]	VIC	2.5	188–219	28	0.947	0.924	0.005
rw48	Waldick *et al.* [40]	NED	3	108–133	10	0.882	0.500	0.026

### 2.3. Hormone extraction and quantification

To develop an assay for pregnancy, we extracted progesterone from the blubber portion of the biopsy samples following standard methods [44,45]. A cross-sectional subsample (approx. 0.15 g) spanning from the epidermis–blubber interface to the most internal layer of the biopsy was subsectioned. These subsamples were then homogenized multiple times using an automated, multi-tube homogenizer (Omni International). Following the completion of the homogenization process, progesterone was isolated using a series of chemical washes, evaporations and separations. The final hormone residue was stored at −20°C until analysis. The amount of hormone in each extract was quantified using a commercially available enzyme immunoassay. Before analysis, samples were re-suspended in phosphate-buffered saline and then assayed. The progesterone enzyme immunoassay kit (EIA kit 900-011, ENZO Life Sciences, Farmingdale, NY) used in this study has 100% reactivity with progesterone and an assay detection limit between 15 and 500 pg ml^−1^. Two additional standard dilutions were added to allow for a lower detection limit of the standard curve to 3.81 pg ml^−1^. If reliable hormone concentrations were not obtained during the initial assay process, extracts were further diluted and re-run.

As part of our routine quality control, we determined the extraction efficiency by spiking subsamples of blubber from a dead animal with the target hormone [44]. The percentage of hormone recovered after the extraction was calculated, and each sample concentration was adjusted to this efficiency before statistical analyses. An extraction efficiency greater than 60% was acceptable. If the extraction efficiency was less than 60%, the sample extracts were discarded, and the blubber samples re-extracted. Additionally, we conducted a parallelism test to gauge the performance of using the AMW blubber extracts with the progesterone EIA kit. This was done by taking a serially diluted pool of sample extracts and running them along with the standard controls of the assay to determine whether the linear decrease in measured values of the pooled sample was parallel to the standard curve. This would indicate that the assay measures the same antigens in the blubber as in the standards. Extracts from six individual females were pooled together, and the pooled sample concentrations were made by diluting five times from the neat preparation to 1/32, decreasing by a factor of two. Each dilution was run two times, and the resulting curve of the concentrations as a function of the mean optical density was then compared with the standard curve.

### 2.4. Pregnancy classification

**Figure 2.**
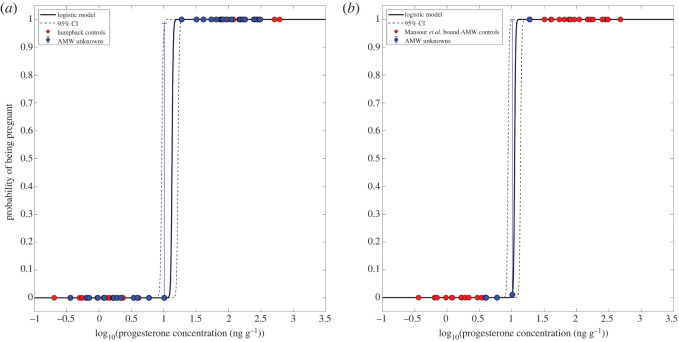
Logistic regression models for the probability of pregnancy in Antarctic minke whales (AMWs) relative to blubber progesterone concentration. (*a*) Model based on the relationship of AMW progesterone concentration with a reference model developed from known pregnant humpback whales adapted from Pallin *et al*. [46] and (*b*) Mansour *et al*. [47] adapted model built by bounding sampled AMWs according to common minke whale progesterone concentrations. Red circles represent mature female humpback whale females (*a*) and mature female AMWs (*b*) used to develop the model. Blue circles represent the AMW females of unknown pregnancy status sampled along the Western Antarctic Peninsula with an associated error around their probability of pregnancy. Dashed lines represent the 95% confidence envelopes developed from 10 000 bootstrap iterations. x-axis values are log_10_ transformed.

To assign pregnancy in sampled AMWs, we adapted two methods used by Pallin *et al*. [46] for humpback whales (*Megaptera novaeangliae*). Similar distributions in the progesterone concentrations were observed for both humpback whales and AMWs and as such, we first assigned the pregnancy status of female AMWs based on the relationship of their progesterone concentration with a reference model developed from known pregnant humpback whales [46]. We then used the range in concentrations of progesterone from female common minke whales (*Balaenoptera acutorostrata*) of known pregnancy status as described in Mansour *et al*. [47] to build a second model from our sampled AMWs which fell within those bounds. Specifically, a gap in progesterone concentrations was observed between a maximum of 3.43 ng g^−1^ in not-pregnant females and a minimum of 22.84 ng g^−1^ in pregnant animals, with an almost 60-fold difference observed between the mean blubber progesterone concentrations among these two pregnancy state designations. For the AMW samples which fell between the ranges for not-pregnant and pregnant common minkes (*N* = 5), we interpreted their pregnancy state based on the relationship of their progesterone concentrations with the reference levels from the second model. In both cases, the models determined the probability of pregnancy (point estimate) and 95% confidence envelope. Using both the point estimate and associated error, we were then able to assess confidence of the pregnancy assay (e.g. >99.9% is pregnant, <0.1% not-pregnant, 0.1% < *p* < 99.9% undetermined (UND)) [46]. Moreover, we were also able to provide an estimate of the proportion of pregnant females (pregnancy rate) in all samples, including those with an assignment probability between 0.1% and 99.9%. This was accomplished by taking the sum of the probabilities for all samples at each individual model bootstrap replicate and dividing by the sample size to obtain the proportion pregnant. The resulting pregnancy rates from each model were compared with each other (figure 2).

### 2.5. Unoccupied aircraft systems image collection and photogrammetry

To determine the distribution of size classes of AMWs in the population, high-resolution aerial images were collected using unoccupied aircraft systems (UAS, or drones) and analysed to estimate the total length of AMWs. These images were collected during the 2017–2019 austral summer (January–March) field seasons using three different hexacopters: APH-22 (Aerial Imaging Solutions), Alta 6 (FreeFly) and LemHex-44 (Mikrokopter). The APH-22 was fitted with an Olympus E-PM2 camera with a Micro Four Thirds (17.3 × 13 mm) sensor, 4608 × 3456 pixel resolution, and an Olympus M. Zuiko 25 mm f1.8 focal length lens. Both the LemHex-44 and Alta 6 were equipped with a Sony Alpha a5100 camera with an APS-C (23.5 × 15.6 mm) sensor, 6000 × 4000 pixel resolution and either 35 or 50 mm f1.8 Sony SEL focal length lens. Each aircraft had an onboard barometer, while the LemHex-44 and Alta 6 were also equipped with a LightWare SF11/C laser altimeter to determine the altitude of each image. Details for flight operations and image collection are described in Kahane-Rapport *et al*. [48] for the Alta 6 and LemHex-44 and in Durban *et al*. [49] for the APH-22. Individuals were identified from external marking and pigmentation patterns that were visible in the aerial and/or boat-based photo-identification images.

**Figure 4.**
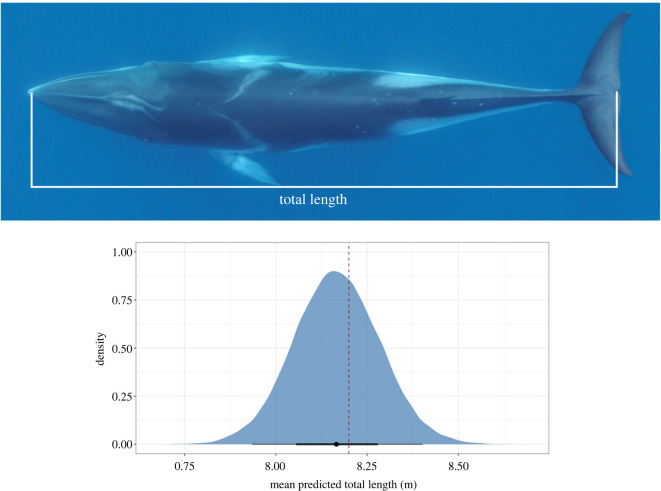
An example of a UAS image of an Antarctic minke whale (AMW). Total length is measured from the tip of the rostrum to the fluke notch. The bottom panel shows an example of a posterior predictive total length distribution for a single individual. The longer black bars represent the 95% highest posterior density (HPD) intervals, the thicker shorter black bars represent the 65% HPD interval, and the black dot represents the mean value. The red dashed line represents the median length at sexual maturity for female AMWs generated from commercial catch data (8.20 m).

Images were selected for each individual and ranked for quality in measurability following Christiansen *et al*. [50], in which a score of 1 (good quality), 2 (medium quality) or 3 (poor quality) was applied to seven attributes: camera focus, straightness of body, body roll, body arch, body pitch, total length measurability and body width measurability. Images with a score of 3 in any attribute were removed from the analysis, together with any images that received a score of 2 in both roll and arch, roll and pitch or arch and pitch [50]. Measurements from up to five images were used per individual. We used MorphoMetriX open-source photogrammetry software to measure (in pixels) total length, from the tip of the rostrum to the fluke notch (figure 4) [51]. MorphoMetriX outputs were collated using CollatriX open-source software [52].

**Figure 3.**
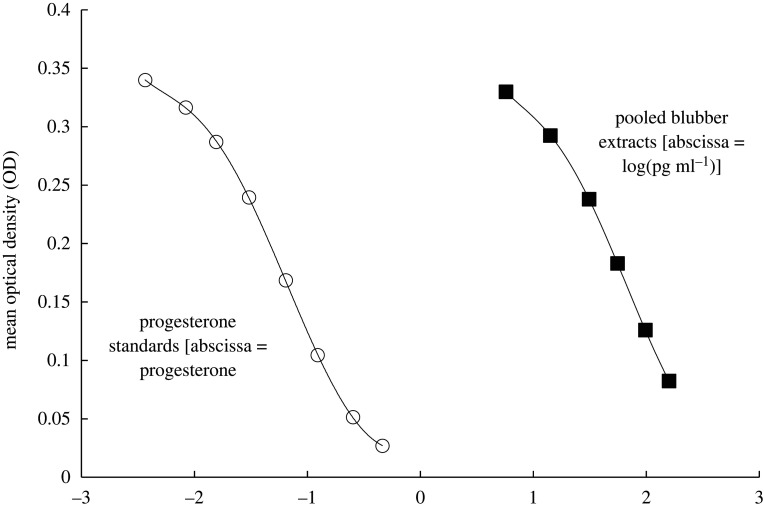
Results from linearity assessment of progesterone enzyme immunoassay (EIA) with Antarctic minke whale (AMW) blubber tissue extracts. Serial dilutions of extracts (shaded squares) show parallelism with the standards of the progesterone EIA (open circles) (*r*^2^ = 0.982, slope = 0.997); an indication that the assay is measuring the same antigens in the blubber as in the standards and therefore suitable for use with AMW blubber tissues extracts. Six individual females were represented in the pooled blubber extracts.

To account for measurement uncertainty associated with each UAS, we used the Bayesian statistical model described in Bierlich *et al*. [53], in which training data of known-sized objects measured at various altitudes are used to predict length measurements and associated uncertainty of objects of unknown size (e.g. each individual whale). For the Alta 6 and LemHex-44, we employed the dataset used by Bierlich *et al*. [53] for training the model with measurements from images of known-sized floating objects (*n* = 110) collected between 10 and 120 m altitude along the WAP (length = 1.33 or 1.40 m), Monterey, California (length = 1.27) and Beaufort, North Carolina (length = 1.48). For the APH-22, we used images of the rail on the rigid-hull inflatable boat (RHIB; length = 2.95 m) collected at altitudes of 22–47 m as training data. The training data encompassed the range in altitude that images of AMWs were collected for each aircraft (Alta 6 and LemHex-44 : min = 15, max = 83 mean = 42.30, s.d. = 16.92; APH-22; min = 30, max = 42, mean = 36.67, s.d. = 2.85). Rather than a single-point estimate, the model generated a posterior predictive distribution for the total length (m) of each individual (figure 3). We then estimated the total length of each individual as the mean of its posterior predictive distribution and assessed measurement uncertainty by constructing the 95% highest posterior density (HPD) intervals, which is an interval that represents the region with a 95% probability of encompassing the parameter of interest (e.g. total length; figure 4). Model development and analyses were conducted in R (v. 3.6.1 [54]), as described in Bierlich *et al*. [53].

### 2.6. Estimating proportion of mature females using an undifferentiated sample of animals of known length

If length, sex and pregnancy data were available from the same individual AMW, it would be possible to estimate the length at maturity directly and use this to calculate the total number of mature animals in the sample. However, this was not possible for AMWs imaged in this study, and as such, the pregnancy rate presented here was based solely on hormonal estimates, and these data do not distinguish between sexually immature and mature not-pregnant females. Unfortunately, sex and maturity status were not available for our sample of whales whose lengths have been estimated via UAS imaging. However, the lengths of whales can be used to estimate the expected proportion of mature females in our sample if the probabilities that an animal of a given length is both female and mature can be calculated from other sources. This is possible for the Antarctic Peninsula region using data from historical commercial whaling catches (1972–1987) in the region bounded by the longitudes 55° W to 70° W (data provided by International Whaling Commission (IWC) archives). It is not necessary that the distributions of lengths in our samples and from the commercial data are comparable, only that the proportions of animals that are female and mature are similar at a given length. The methods for estimating the sex ratios and maturity status at given lengths are described in electronic supplementary material, Appendix I. These calculations include Bayesian estimates of the sex ratio and maturity at-length distributions, and consequently, the distributions of the sex ratio and pregnancy rates based on our length and pregnancy data can be estimated.

### 2.7. Data preparation and statistical analyses

We used a two-tailed exact binomial test [55] to test for deviations from a 1 : 1 sex ratio (parity) across the entire dataset and within a given year. Additionally, to avoid re-sample bias in our analyses, we removed all within-year replicates. In both cases, the most recent sample was retained for the analyses. For all statistical tests, we considered a *p*-value of less than 0.05 to be significant. All values are expressed as mean ± s.d. unless otherwise stated.

## 3. Results

### 3.1. Individual identification and sex

We collected 82 skin and blubber biopsy samples along the WAP from 2013 to 14 and 2016 to 20 (figure 1). Samples were collected from January to July, with most (64%) collected in February. An average of 9.4 microsatellite loci were successfully genotyped per individual. Fifteen samples failed the initial genotype quality control and were re-analysed. Three samples (2013, one male, one female; 2018, one male) never yielded a high-quality genotype and were not included in further analyses. The average *P*_ID_ for any given combination of seven loci ranged from 5.74 × 10^–14^ to 1.57 × 10^–11^, consistent with similar studies [45]. DNA profiling was sufficient to identify and determine the sex of 69 individual whales from these samples (table 2). In total, we sampled 29 individual males and 40 individual females throughout the study. Details on annual sampling can be found in table 2. We resampled nine individuals within the same year. Five individuals were resampled on the same day (2014, two females; 2020, two males, one female), including one female that was sampled three times (2014, one female). Additionally, one individual was resampled one day apart (2017, one male), one individual was sampled three days apart (2013, one female), one individual was sampled six days apart (2019, one female) and one individual was sampled 24 days apart (2020, one male). We did not recapture any individuals across years. Overall, we sampled more females than males (0.73 M : F), but this deviation from parity was not significant (*p =* 0.228, exact binomial test, table 2). In addition, the sex ratios did not differ from unity in any sampling year (table 2).

**Table 2.** Sample summary statistics for Antarctic minke whales sampled along the Western Antarctic Peninsula (2013–14, 2016–20) with known genetic sex. † Designates that all replicates in the dataset have been removed (i.e. this is the true number of individuals in the dataset). *^β^* Does not include samples that yielded a poor genotype quality score (2013, one male, one female; 2018, one male).

temporal scale	*N*	# genotypes	male		95% CL	female		95% CL	sex ratio	difference to parity	pregnant (females only)		
			*N*	*%*	lower–upper	*N*	*%*	lower–upper	(M : F)	exact binomial test	*N_ind. analysed_*	*N_pregnant_*	%
2013 total	19	16*^β^*	5^*β*^	31.25	11.02–58.66	11*^β^*	68.75	41.34–88.98	0.45	*p* = 0.210	9	6	66.67
2014 total	10	7	1	14.29	0.36–57.87	6	85.71	42.12–99.64	0.17	*p* = 0.125	4	3	75.50
2016 total	5	5	3	60.00	14.66–94.73	2	40.00	5.27–85.34	1.50	*p* = 1.000	2	2	100.00
2017 total	10	9	6	66.67	29.93–92.51	3	33.33	7.49–70.07	2.00	*p* = 0.508	3	3	100.00
2018 total	8	7*^β^*	3^*β*^	42.86	9.90–81.59	4	57.14	18.41–90.10	0.75	*p* = 1.000	3	2	66.67
2019 total	14	13	4	30.77	9.09–61.43	9	69.23	38.57–90.90	0.44	*p* = 0.267	9	2 (1 UND)	22.22
2020 total	16	12	7	58.33	27.67–84.83	5	41.67	15.17–72.33	1.40	*p* = 0.774	4	2	50.00
total	82	69†	29	42.03	30.24–54.52	40	57.97	45–47–69.76	0.73	*p* = 0.228	34	20	58.82

### 3.2. Variation in progesterone concentrations

Based on the concentrations observed from a series of spiked controls, our average extraction efficiency for the progesterone assay was 82.77% ± 14.46 (minimum 65.78%, maximum 100.81%). Additionally, our calculated intra-assay and inter-assay coefficient of variation from a series of replicated samples was 3.10% and 7.21%, respectively. The EIA standards and the pooled serially diluted blubber extracts exhibited statistical parallelism (figure 3, *r*^2^ = 0.982, slope = 0.997); an indication that the assay is measuring the same antigens in the blubber as in the standards and therefore is suitable for use with AMW blubber tissues extracts.

We measured progesterone concentrations in 39 samples obtained from 34 individual female AMWs (figure 1). A small number of samples were excluded from the analysis due to within-year re-sampling, insufficient blubber for extraction, or a poor-quality genotype. In both probability assignment methods, 13 individual females were estimated to have a probability of being pregnant of less than 0.1% (assigned as not-pregnant; *p* < 0.1%; blubber progesterone: mean = 1.98 ± 1.58 ng g^−1^) and 20 were estimated to have a higher than 99.9% probability of being pregnant (assigned as pregnant, *p* > 99.9%; blubber progesterone: mean = 144.86 ± 96.53 ng g^−1^; table 3; figure 2). Additionally, one individual whose progesterone concentrations fell within the 95% confidence envelope in both models (blubber progesterone: 10.20 ng g^−1^), received a mean probability of being pregnant of 0.53%, with a lower CI of 0.00% and an upper CI of 99.30%; table 3; figure 2). This individual received an undetermined pregnancy assignment. The mean estimated proportion of pregnant females, across both models, across all 34 samples was 58.84% (CI = 58.82–61.74%). The within-year replicate samples provided further validation of the assay by demonstrating that re-sampled females continued to fall within the same pregnancy designation. Specifically, two females were consistently classified as not-pregnant (gBbo19AP006: mean = 3.43 ± 0.68 ng g^−1^, six days between resampling; gBbo20AP08: mean = 2.48 ± 2.16 ng g^−1^, sampled same day) and two females as pregnant (gBbo14AP001: mean = 391.66 ± 123.36 ng g^−1^, sampled same day; gBbo14AP005: mean = 110.60 ± 99.39 ng g^−1^, sampled same day). Lastly, the distribution in progesterone concentrations across our two designated pregnancy states for female AMWs sampled along the WAP was distributed similarly to common minke whales as outlined in Mansour *et al*. [47], as well as to samples collected from female humpback whales also sampled along the WAP [45].

**Table 3.** Progesterone concentrations (ng g^−1^) of presumed pregnant and not-pregnant Antarctic minke whales biopsied along the Western Antarctic Peninsula. *^β^* Does not include samples that yielded a poor genotype quality score (2013, one female).

	mean (ng g^−1^)	s.d.	Min	max	*N*
not-pregnant	1.98	1.58	0.36	5.92	13
pregnant	144.86	96.53	18.85	307.01	20^*β*^
undetermined	10.20				1
total					34*^β^*

### 3.3. Group compositions

The group composition of AMWs sampled throughout this study varied from single animals, pairs, to large aggregations of up to 25 individuals. Of the whales sampled with reliable sighting data in this study, 24 were encountered as singletons (16 M and 8 F), seven were found in pairs and 36 were found in groups of three or more. Additionally, we fully sampled all individuals present in four groups, including two pairs (FF, MF), one group of three (MFF) and one group of four (MMMM). No calves were observed during this study.

### 3.4. Length frequencies

**Figure 5.**
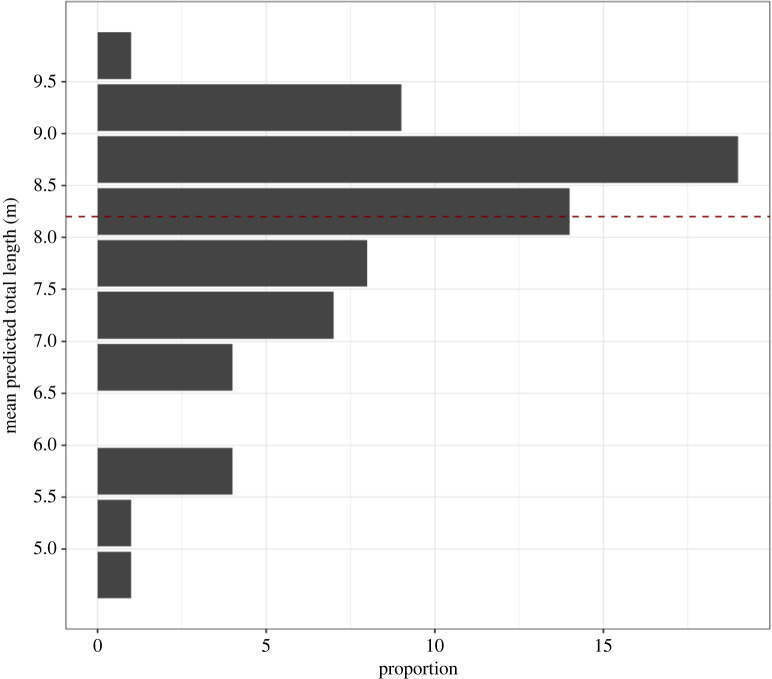
Length frequency distribution of Antarctic minke whales (AMWs, *N* = 68) with the 95% highest posterior density (HPD) interval. The total length of each individual is represented by the mean of its predicted posterior total length distribution (figure 4). The dashed line represents the median length at sexual maturity for female AMWs generated from commercial catch data (8.20 m).

A total of 68 AMWs were photographed by UAS along the WAP between January and March during the 2017–2019 field seasons (figures 1, 4, 5; mean = 8.04 m, s.d. = 1.06, min = 4.65, max = 9.74). Measurement uncertainty, measured as the width (m) of the 95% HPD interval for each individual (figure 4), was similar across each UAS aircraft: mean = 0.45, s.d. = 0.28, min = 0.15, max = 1.55. No individual was measured more than once during the study period, nor did we observe any behavioural responses of the AMWs toward the UAS.

### 3.5. Adjusted demographic parameters using commercial catch data

**Figure 6.**
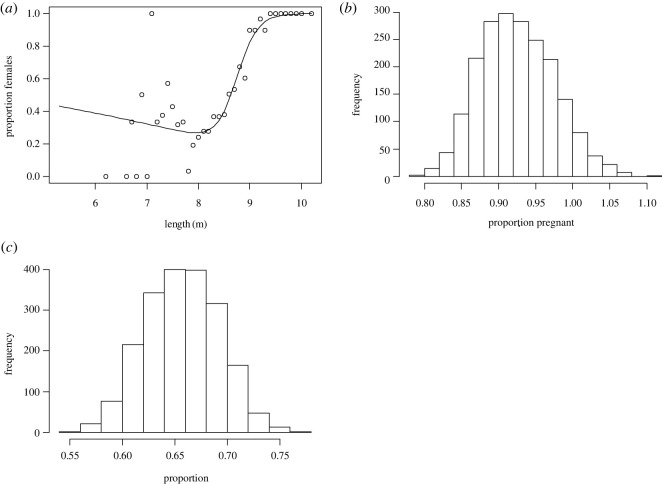
(*a*) Proportion of Antarctic minke whale females at length from commercial catch data in the vicinity of the Antarctic Peninsula (points) and the maximum-likelihood estimate of the product of two logistic functions (line) and the posterior distribution of pregnancy rates (*b*) and proportion of mature females (*c*) derived from the observed lengths using the Monte Carlo Markov chain (MCMC) calculations of the proportions of mature females at given lengths derived from commercial catch data (see electronic supplementary material, Appendix I).

Applying the proportions of sex at length from the catch data to our length data provides a median value for the sex ratio of 1.04 M : F (49% female, 95% credible interval 44%–54%). Figure 6*a* shows the maximum-likelihood estimate from the commercial catches of the probabilities at each length that an animal is a mature female. The median estimate of the female length at 50% sexual maturity from the catch data was 8.20 m (95% credible interval 8.10–8.28; electronic supplementary material, Appendix I). Applying the product of this curve with the maximum-likelihood estimates of the commercial catch length at maturity to our length data gives the proportion of females that are mature as 66.5%. Thus, the maximum-likelihood estimate for the number of mature whales in our sample of 33 females is 22. Twenty of the known sampled females were pregnant and hence the estimated pregnancy rate of adult females is 90.09% under the assumption that the length frequency distribution of the UAS-measured animals is the same as the unknown length frequency distribution of the biopsied animals. Uncertainty in the estimates of the proportion of mature females was calculated using a Monte Carlo Markov chain (MCMC) analysis (see electronic supplementary material, Appendix I) for the two functions. This produced the distribution for pregnancy rate shown in figure 6*b*. This distribution had a median pregnancy rate of 92.3% (95% credible interval 83.8%–102.8%). The distribution has a tail above 100%, reflecting the uncertainty in the estimated proportion of mature females as shown in the distribution given in figure 6*c*.

## 4. Discussion

Our results provide the first estimates of sex ratio, maturity status and pregnancy rates for Antarctic minke whales (AMWs) inhabiting the waters surrounding the Western Antarctic Peninsula (WAP), a region undergoing rapid environmental change. Our comparisons may have more uncertainty than we can account for as we adjusted our data using data collected during commercial whaling operations that occurred 40 years earlier. This study also represents the first demographic study of this species using non-lethal techniques.

### 4.1. Variation in sex ratios

The sex ratio of the sampled population was biased towards females (0.73 M : F), but this was not statistically different from parity. In addition, the Bayesian estimated sex ratio for this region was 1.04 M : F. Similar sex ratios (mean: 1.18 M : F, range: 0.48–3.26) were observed among 4383 individual AMWs lethally sampled between 1990 and 2006 in East Antarctica [56]. Sex ratio biases occur in other parts of the Antarctic that seem to be related to latitude: in a study of minke whales killed under a Special Permit whaling programme, females represented 80% of the catch near the ice edge, but males dominated in waters north of 65° S, further from the ice edge [57]. Similarly, skewed sex ratios have been observed as a result of demographic segregation in common minke whales off Greenland [58]. Population segregation is found in a wide variety of species [59]. In cetaceans, spatial sex segregation is broadly observed [60,61], with adaptive advantages to social structure, environmental constraints, niche selection and timed with life-history events. Our samples come from a small region of the WAP relative to the range of the species in this region [21]. AMWs satellite-tagged in our study area maintained a coastal distribution along a large latitudinal gradient that affords them coastal shelter and proximity to available sea ice [21,23]. If there is spatial segregation related to distance from shore or ice shelf (i.e. sea ice versus open water), then our sample population could be skewed toward females, which show a strong affinity toward structure in other areas of the Antarctic.

### 4.2. Variation in pregnancy rates

We calculated an unadjusted pregnancy rate of 58.84% for all females sampled during this study and a corrected pregnancy rate (including only sexually mature females) of 92.3%, which is similar to East Antarctica (mean 90%) [62]. It is likely that the high abundance of krill along the WAP [63,64] in combination with AMWs' unique ecological niche [24] supports the high observed reproductive rates within this population. Similar high rates of pregnancy among other baleen whales in this region have also been attributed to the high productivity of the WAP [45] and lack of other baleen whales that have yet to recover from commercial whaling (i.e. blue and fin whales). Unfortunately, it is not possible to reconstruct the demographic trajectory of these whales over the last half-century because there is no good historical baseline for this population. However, continued demographic monitoring will allow us to understand how this population is responding to climatic change which will probably lead to a decline in the amount of physical habitat and prey available to them over time [23,64].

### 4.3. Variation in length frequencies and sexual maturity

Our overall mean length of AMWs was 8.04 m and we estimated that 66.5% of females were sexually mature. Both our mean calculated length and the proportion of presumed sexually mature female AMWs from this study were lower than the reported mean length (8.59 m) [57] and reported proportion of sexually mature female AMWs (76%) [27] killed under the Special Permit whaling programmes. This is not surprising given that these whaling programmes may have been biased toward catching larger individuals near the ice edge [65]. The East Australian humpback whale, a closely related long-lived species, has a high proportion of young and sexually immature individuals, indicative of a rapidly growing population [66]. Continued monitoring of AMWs along the WAP is needed to better understand their age structure in the context of a rapidly changing environment.

### 4.4. Potential effects of spatial and temporal heterogeneity

In the present study, we sampled whales opportunistically and avoided re-sampling individuals whenever possible. The distribution of AMWs is segregated by sex, age and reproductive status. Specifically, in the East Antarctic, immature whales are normally solitary and occur in lower latitudes further offshore. Mature males are more abundant in middle latitudes, and mature females occur in greater frequency in higher latitudes near the marginal pack ice zone [57]. For the subset of whales that migrate for reproductive purposes, males tend to arrive in the Antarctic in November, with females on average arriving one month later as a result of weaning their calves in lower latitudes [67] and then remain in the region. By February, mature females dominated 85% of the catch south of 65 degrees [57]. Mature females' high affinity for the ice zones may make them susceptible to changes in their environment, and as a result they are likely to be the best indicators of changing population dynamics. Although data on the spatial and temporal segregation of AMWs comes from East Antarctica, it is possible that similar spatial and temporal dynamics exist for this species along the WAP, and that our results reflect a similar distribution pattern. Almost two-thirds of our tissue samples and 50% of our UAS images were collected in February, and spatially, our sampling was focused within a subset of the known range of AMWs in this region. To better understand these potential biases, a more systematic and comprehensive spatio-temporal sampling effort is required. For example, we suggest future work to pair remote biopsy sampling and UAS imaging of individual whales across the entire continental shelf during a more protracted summer season. Finally, this study has successfully demonstrated the ability to assess the length, sex, maturity and pregnancy status of AMWs sampled non-lethally, and these methodologies can now be employed in more comprehensive, long-term studies.

### 4.5. Climate change effects on population dynamics of Antarctic minke whales

The rates of population decline in birds and mammals globally are greater in locations where the temperature has increased at higher rates [68]. The marine environment of the WAP is experiencing some of the most significant warming on Earth, resulting in a rapidly diminishing extent and duration of sea ice. Taken together, these climatic changes represent some of the most dramatic changes in the physical environment on the planet [29]. The distribution and ecology of AMWs are directly tied to sea ice and prey availability, and changes that impact both the quantity and quality of their habitat and food availability may result in significant effects on fitness. We are already witnessing temporal contraction of critical habitat for AMWs in this region [29], as shown by satellite tracking data [21]. The WAP population of humpback whales, which is growing at rates at or near maximum values [45], and AMWs partition prey by feeding in different habitats (sea ice versus open water, and vertically in the water column) [69], but with continued declines in sea ice, the ability to successfully partition foraging habitat may also decline, and competition for prey could increase between the two species. If AMWs are forced to broaden their distribution to suboptimal areas, they would be at higher predation risk from Type A killer whales in open water [26]. Similar population-level responses among other ice-dependent krill consumers along the WAP have been documented in response to environmental change. Over the last 50 years, Adélie penguin populations have decreased dramatically and ice-intolerant chinstrap and gentoo penguin populations have increased substantially [70]. In the light of the better-documented population responses of penguins to changes in physical substrates, such as sea ice, it is not implausible that WAP marine mammal populations (e.g. AMWs and killer whales [71,72]) that are similarly associated with these variables have responded or will respond in the same way.

## 5. Conclusion

Our study provides the first data on the demographics and population structure of AMWs along the WAP and the first non-lethal study of the demography of this species. Our results provide key information for the population status of AMWs in a rapidly changing system. As the extent of seasonal sea ice and krill continues to decline around the WAP, AMWs may become displaced by lack of preferred habitat and/or increasingly susceptible to competition and predation, as has been observed in other baleen whales [73] and ice-obligate marine predators [32].

## Data Availability

The datasets supporting this article have been uploaded as part of the electronic supplementary material [74].
